# Dairy Product Consumption and Prostate Cancer Risk in the United States

**DOI:** 10.3390/nu11071615

**Published:** 2019-07-16

**Authors:** Isabella Preble, Zhenzhen Zhang, Ryan Kopp, Mark Garzotto, Gerd Bobe, Jackilen Shannon, Yumie Takata

**Affiliations:** 1 College of Public Health and Human Sciences, School of Biological and Population Health Sciences, Oregon State University, Corvallis, OR 97331, USA; 2 Knight Cancer Institute, Oregon Health and Science University, Portland, OR 97239, USA; 3 Department of Urology, Oregon Health and Science University, Portland, OR 97239, USA; 4 Linus Pauling Institute, Oregon State University, Corvallis, OR 97331, USA

**Keywords:** dairy products, prostate cancer risk, United States

## Abstract

An ongoing controversy exists regarding the effect of dairy products on prostate cancer risk in observational studies. We prospectively investigated the associations between dairy product consumption and prostate cancer risk among men in the United States. After calculating pre-diagnostic intake of individual or subgroups of dairy products using a validated food frequency questionnaire, we estimated hazard ratios (HR) and 95% confidence intervals (CI) for pathologically-verified cases of incident prostate cancer among men, overall, or stratified by severity. Among 49,472 men, 4134 were diagnosed with prostate cancer during an average follow-up period of 11.2 years. The median total dairy intake was 101 g/1000 kcal. Consumption of total, individual, or subgroups of dairy products was not statistically significantly associated with prostate cancer risk overall (HR = 1.05, 95% CI = 0.96–1.15 comparing the highest with lowest quartile) or stratified by severity, except for regular-fat dairy product intake with late-stage prostate cancer risk (HR = 1.37, 95% CI = 1.04–1.82 comparing the highest with lowest quartile) and 2%-fat milk intake with advanced prostate cancer risk (HR = 1.14, 95% CI = 1.02–1.28 comparing the higher than median intake with no intake group). Our findings do not support the previously reported harmful impact of dairy consumption on overall prostate cancer risk among men in the United States.

## 1. Introduction

Dairy products contain many nutrients and bioactive compounds linked to human health [1] and vary widely in fat content as well as processing methods, such as fermentation. For instance, high saturated fatty acid content in some dairy products has been implicated in the etiology of many chronic diseases, including cancer [2]. Fermented dairy products have been linked to the promotion of growth of intestinal microbiota that prevent chronic diseases, including cancer [3,4], and provide lactose-free alternatives to allow for the intake of minerals and vitamins high in dairy products for lactose-intolerant individuals. Hence, it is important to investigate dairy products separately by fat content and fermentation method. In most previous epidemiological studies, their food frequency questionnaires (FFQs) had few questions to assess dairy product intake by fat content (other than milk) and fermentation method.

Among chronic diseases, prostate cancer, the most common non-skin cancer and the second leading cause of cancer-related deaths among men in the United States (US) [5], is an interesting area of research when examining dairy products; an ongoing controversy exists regarding the effect of dairy intake on prostate cancer risk in observational studies. The Dietary Guidelines for Americans 2015–2020 recommends lower fat options of dairy products [6], although positive associations of prostate cancer risk with low-fat milk intake and an inverse association with whole milk consumption have been reported in the most recent meta-analysis of prospective studies, the strongest study design in observational studies [7]. This meta-analysis is included as the latest summary of dairy products and prostate cancer risk in the latest World Cancer Research Fund and American Institute for Cancer Research expert report [8]. Since the publication of this meta-analysis, no additional prospective studies have been published on risk of incident prostate cancer and dairy product intake during middle to late adulthood. The summary relative risk (RR) and 95% confidence intervals (CI) for the highest compared with the lowest intakes was 1.14 and 1.05–1.25 for low-fat milk. Conversely, there was a significant inverse association between whole milk and prostate cancer risk (RR = 0.92, 95% CI = 0.85–0.99 comparing the highest with lowest intake category). In addition, total dairy product, total milk, and total cheese intakes were significantly positively associated with prostate cancer risk (total dairy products: 1.09, 1.02–1.17; total milk: 1.11, 1.03–1.21; and total cheese: 1.07, 1.01–1.13), while consumption of yogurt, skim milk, ice cream, and butter had no significant associations with prostate cancer risk.

This meta-analysis was limited by an insufficient number of previous studies that investigated associations by severity, preventing a stratified analysis [7]. Of the individual previous studies reporting associations by severity, the results were heterogeneous [7]. Furthermore, a relatively limited number of studies reported associations by individual or subgroups of dairy products and found statistically significant positive associations with whole milk and cheese, but heterogeneous results for low-fat milk and yogurt [7]. It is important to investigate associations in subgroups or by individual products, given that potential biological effects of dairy on carcinogenesis may differ by product. For example, lactose enhances calcium absorption [9], which affects calcium and vitamin D levels, both of which may affect prostate cancer risk [10]. Animal fat is hypothesized to promote prostate carcinogenesis by increasing testosterone levels [11], consequently activating pro-oncogenes and deactivating tumor suppressor genes [12]. Fermented dairy products may affect prostate cancer risk through their effects on intestinal microbiome [13]. Milk intake may increase proliferation of cancer cells through elevated insulin-like growth factor-I (IGF-1), which is linked to an increased risk of prostate cancer. In a recent meta-analysis by Harrison et al. [14], 51 studies supported this hypothesis, and another meta-analysis of 12 prospective studies reported a 38% increase in prostate cancer risk with high concentrations of IGF-1 [15].

In this analysis, we investigated associations between dairy product consumption and risk of prostate cancer using the prospective Prostate, Lung, Colorectal and Ovarian (PLCO) Cancer Screening trial cohort. We examined dairy product consumption as total (all dairy products), by fat content (regular- or low-fat), by fermentation methods (fermented or non-fermented), and milk consumption as total or by fat content (nonfat to whole). We examined all prostate cancer cases and also separately by severity.

## 2. Methods

### 2.1. Study Population

This is a prospective analysis of the PLCO, which is a two-armed, randomized trial of cancer screening intervention with no dietary intervention [16]. The PLCO study not only investigated the effects of cancer screening protocols for early detection of these four types of cancer, but also collected potential etiological factors for various cancers, including prostate cancer. Briefly, the participants included in the current analyses were recruited from ten screening centers (Birmingham, AL; Denver, CO; Detroit, MI; Honolulu, HI; Marshfield, WI; Minneapolis, MN; Pittsburgh, PA; Salt Lake City, UT; St Louis, MO; and Washington, DC) participating in the PLCO trial (ClinicalTrials.gov identifier: NCT00002540) and were between 55 and 74 years at the time of enrollment (1993 to 2001). For participants randomized into the intervention arm, prostate cancer screening through prostate-specific antigen (PSA) and digital rectal examination (DRE) was offered annually for three years. Thereafter, prostate cancer screening through PSA test only was offered for two consecutive years. Participants randomized into the control arm received their usual care, including opportunistic screening requested by a participant or a doctor. All study participants provided written informed consent, the study protocol was approved by institutional review boards at the National Institute of Health/National Cancer Institute (OH97-C-N041), and the current project was approved by the National Cancer Institute Cancer Data Access System (PLCO-224).

Detailed exclusion criteria for enrollment to the PLCO trial were previously described [16]. Inclusion criteria for the current analysis was providing the diet history questionnaire (DHQ), an FFQ, prior to prostate cancer diagnosis. The DHQ was administered at study entry (for participants in the control arm randomized after December 1998) or during the next follow-up after 1998 (for participants randomized before 1998), and at about the first or third year of follow-up (for participants in the screening arm, starting December 1998). DHQ was completed by 77% of the participants in both control and screening arms, and we included participants in both arms as a prospective cohort.

### 2.2. Diet History Questionnaire

To assess dairy product intakes as well as other food item and nutrient intakes, we used a self-administered, validated, 36-page DHQ which evaluated the participants’ usual consumption frequency and portion size of 124 food and beverage items over the past 12 months [17,18]. Related aspects of consumption were also asked for 47 of these items, such as fat content. The DHQ asked a total of 57 questions (not counting questions about mixed dishes) regarding dairy product intake, which included frequency, portion size, fat content, and/or how they were consumed with or without another food (e.g., type of milk consumed as a beverage, or added to cold cereals vs. coffee) for the following dairy products: milk, cream, sour cream, cream cheese, cottage cheese, cheese sauce, other cheeses, yogurt, frozen yogurt, ice cream, and butter. Most of the food items were further broken down by fat content into regular- or low-fat products. Milk was further broken down to whole milk; 2%-fat milk; 1%-fat milk; skim, nonfat, or 0.5%-fat milk; and/or half and half. The frequency of consumption ranged from “never” to “two or more times per day” for foods and to “six or more times per day” for beverages, and three categories of portion size were provided. The amounts of individual food items consumed were estimated by multiplying the frequency by the portion size each time participants reported a particular food item. In the case of mixed dishes and food mixtures, ingredient components were disaggregated into individual food items and the amount of each ingredient was estimated similarly by the multiplication of frequency and portion size [19]. Total intake was calculated based on questions on single food items (e.g., carrots) and mixed dishes (e.g., stew). Using these food item intakes, total energy and nutrient intakes were estimated using US Department of Agriculture food composition database. The DHQ was validated against four 24-h dietary recalls covering the same 12-month period as the DHQ [18]. After accounting for measurement errors, among men, the correlation coefficient of total energy intake between DHQ and dietary recalls was 0.49 and the correlation coefficients of energy-adjusted nutrient intake ranged from 0.57 for protein and vitamin E to 0.83 for thiamin [18].

### 2.3. Cancer Ascertainment

Prostate cancer screenings were conducted at one of the 10 clinical centers within the PLCO, and those with positive screening results (a PSA test result >4.0 ng/mL or suspicious DRE result) were referred to primary healthcare physicians for further diagnostic examinations. During the 13 active follow-up years, prostate cancer incidence and deaths from any cause were ascertained primarily through a mailed annual questionnaire to update cancer diagnosis made in the preceding year. The incident prostate cancer cases reported on this questionnaire were verified with medical records using a standardized abstracting protocol. Detailed information on prostate cancer diagnoses, including clinical stage and Gleason score, were recorded. Both clinical stage and Gleason score were used to classify prostate cancer into advanced or non-advanced status, as described in the Statistical Analysis subsection below. The follow-up process also included periodic linkage of the participants’ files to the National Death Index and notification of vital status by next of kin. The follow-up ended in the 13th year from study entry or on 31 December 2013, whichever came first.

### 2.4. Statistical Analysis

All analyses were conducted by SAS 9.4 (SAS Institute, Inc., Cary, NC, USA). Food and nutrient intakes were adjusted for total energy intake by calculating intakes per 1000 kcal as previously recommended, in order to lessen possible misclassification inherent in self-reported dietary intake data [20]. We created quartiles of dairy product intake variables (gram/1000 kcal) based on the distribution of all participants at baseline. In terms of serving size, one serving of milk is about 240 g. The characteristics of the study participants were described by quartile of total dairy product consumption. To assess associations of dairy product intake variables (refer to Supplementary Table S1 for detailed grouping of dairy products) with prostate cancer risk, we used multivariate Cox regression to estimate hazard ratios (HR) and the corresponding 95% CI for the second to fourth quartiles of dairy product intake, using the first quartile as the reference. To test for a linear trend, we assigned the quartile median dairy product intake and treated it as continuous in the multivariate model. We *a priori* included age, non-Hispanic race/ethnicity, study center, and study trial arm in the model, as they are established risk factors for incident prostate cancer or account for our study design. We considered following variables as potential confounders based on previous publications of diet and prostate cancer in the PLCO study [21,22]: education, frequency of prostate cancer screening during the follow-up time (PSA and DRE, separately and combined), maximum PSA levels during the follow-up time, family history of cancer, smoking history (status and pack-years of smoking), body mass index (BMI), history of diabetes, physical activity engagement, ibuprofen use, dietary lycopene intake, and vitamin E intake. In our multivariate models, the frequency of total prostate cancer screening, maximum PSA levels, and family history of cancer were included because each changed the β–coefficients of the risk estimate for total dairy product intake by at least 10%. To assess potential differences in associations between dairy product intake variables and prostate cancer risk by the severity of prostate cancer, as reported previously [22,23], we conducted analyses stratified by non-advanced (clinical stages I/II and Gleason scores of <7) or advanced status (clinical stages III/IV and/or Gleason scores of ≥7) and also by each of the characteristics alone (clinical stage, early stages I/II vs. late stages III/IV; and Gleason score, low-grade: <7 vs. high-grade: ≥7).

## 3. Results

There were 4134 confirmed prostate cancer cases out of the 49,472 men in the PLCO cohort between 1998 and 2013 during the average of 11.2 follow-up years. As descriptive analysis, after classifying participants into quartiles of total dairy product consumption, the fourth quartile (>194.7 g/1000 kcal) had the most prostate cancer cases, although only slightly (Table 1). Those in the highest quartile of total dairy intake tended to be slightly older, to be non-Hispanic white, to have a personal history of diabetes, to be never or former alcohol drinkers, and to be never smokers, when compared with those in the lowest quartile. The maximum PSA levels tended to be higher in the higher quartiles; however, there was no clear pattern in the frequency of prostate cancer screenings based on PSA and DRE. Similarly, across quartiles of total dairy consumption, no clear pattern was observed in educational attainment, family history of cancer, BMI, ibuprofen use, and physical activity engagement. Those at higher quartiles tended to report lower total energy intake than those in lower quartiles.

Total dairy products had no statistically significant association with prostate cancer risk (HR = 1.05, 95% CI = 0.96–1.15 for highest quartile compared with lowest quartile, *P*-trend = 0.48) (Table 2). Similarly, there were no associations with low-fat dairy products (HR = 1.07, 95% CI = 0.98–1.17, *P*-trend = 0.34) or regular-fat dairy products (HR = 1.05, 95% CI = 0.96–1.15, *P*-trend = 0.90). When we grouped dairy products by fermentation methods, neither fermented dairy products (HR = 1.01, 95% CI = 0.93–1.10, *P*-trend = 0.92) nor non-fermented dairy products (HR = 1.07, 95% CI = 0.98–1.17, *P*-trend = 0.15) had a statistically significant association with prostate cancer risk. Similarly, milk intake variables, as total or separately by fat content, were not associated with a risk of prostate cancer (Table 3). The HR (95% CI) for total milk was 1.06 (0.97–1.15) comparing the highest quartile with the lowest with a *P*-trend of 0.39. By individual milk intake, the HR (95% CI) for comparing the higher than median intake and no intake ranged from 1.00 (0.92–1.09) for skim/nonfat/0.5%-fat milk to 1.06 (0.98–1.15) for 2%-fat milk.

When we stratified cases by advanced or non-advanced prostate cancer (defined by both clinical stage and Gleason score; “non-advanced status”: clinical stages I/II and Gleason scores of <7; or “advanced status”: clinical stages III/IV and/or Gleason scores of ≥7), only 2%-fat milk intake was positively associated with risk of advanced prostate cancer (HR = 1.14, 95% CI = 1.02–1.28 comparing the higher than median intake with no intake group). The association of 2%-fat milk intake by advanced status of prostate cancer did not differ (*P*-difference = 0.35). The rest of the dairy variables were not associated with risk of advanced or non-advanced prostate cancer (Table 4 and Table 5).

When we stratified prostate cancer cases by clinical stage or Gleason scores alone, there were no statistically significant associations of dairy product intake variables with prostate cancer risk (Supplementary Tables S2 and S3), except for regular-fat dairy products and late-stage prostate cancer. Regular-fat dairy products had a significant positive association with late-stage prostate cancer (HR = 1.37, 95% CI = 1.04–1.82, *P*-trend = 0.02), but not with early-stage prostate cancer (HR = 1.02, 95% CI = 0.93–1.12, *P*-trend = 0.54). There was no statistically significant association of low-fat dairy product intake with either early-stage or late-stage prostate cancer.

## 4. Discussion

In this prospective analysis of the PLCO study among middle-aged and elderly US men, intakes of total dairy products or dairy products separately by fermentation methods were not statistically significantly associated with a risk of prostate cancer. When we stratified prostate cancer by severity, our study found a statistically significant positive association between 2%-fat milk consumption and risk of advanced prostate cancer; however, the associations did not differ between advanced and non-advanced prostate cancer. Similarly, the consumption of regular-fat dairy products was positively associated with risk of late-stage prostate cancer; however, the associations did not differ by clinical stage. There were no statistically significant associations of other dairy product intake variables with prostate cancer risk stratified by advanced status, clinical stage, or Gleason scores.

The recent meta-analysis reported a statistically significant positive association between total dairy product consumption and prostate cancer risk based on 15 previous prospective studies, however, with a small risk estimate (summary RR = 1.09, 95% CI = 1.02–1.17 for the highest intake category compared with the lowest) [7]. By individual studies included in the meta-analysis, most studies reported no association, which is consistent with our study. Inconsistent with our null finding, a statistically significant positive association between total dairy product intake and prostate cancer risk was reported in three previous studies: the Alpha-Tocopherol, Beta-Carotene Cancer Prevention (ATBC) study [24]; the National Health and Nutrition Examination Survey (NHANES) Epidemiologic Follow-up Study cohort [25]; and the National Institute of Health-American Association of Retired Persons (NIH-AARP) Diet and Health study [26]. The ATBC and NHANES studies [24,25] included many prostate cancer cases who were diagnosed prior to PSA screening becoming common and, hence, clinical relevance may differ from studies that were conducted more recently. For the more recent NIH-AARP study [26], as the publication reported all common cancers, adjustment variables for the association between dairy products and prostate cancer did not include all risk factors for prostate cancer risk. Furthermore, the ATBC study included butter in the dairy product intake [24], while most previous studies did not [27,28,29]. Similarly, the average intake of total dairy products across these three studies varied from 0.6 servings/1000 kcal [26] to 272.8 g/1000 kcal (which is equivalent to slightly above 1 serving of milk) [24] and our median intake was at the lower-end of this range (101.3 g/1000 kcal). Thus, these differences might have contributed to discrepant findings, as well as differences in disease severity and potential differences in dairy products included and their fat content, which we explored further in the following.

When we stratified prostate cancer by advanced status based on both clinical stage and Gleason scores, we observed no statistically significant associations with dairy product consumption, except for 2%-fat milk consumption. There was a statistically significant positive association of 2%-fat milk consumption only with advanced prostate cancer, but not when classified by either clinical stage or Gleason scores alone. Our finding is inconsistent, to some extent, with two previous studies; one which reported a positive association between skim/low-fat milk and risk of early-stage prostate cancer [28], not advanced prostate cancer; and the other which reported an inverse, not positive, association between total milk and aggressive prostate cancer [30]. Nonetheless, our observed positive association is consistent with the hypothesized link between milk and IGF-1 levels [14], although the link between milk and IGFs has not been reported as fat-content specific. We did not find statistically significant associations of prostate cancer overall or by severity with total milk intake or other individual milk intake by fat content. In the case-control study nested within the screening arm of the PLCO, IGF-1 levels were not associated with prostate cancer risk [23], not giving credence to the hypothesized link between IGF-1 and prostate cancer in our study population. Hence, our finding on the 2%-fat milk intake may be due to chance. Given that relatively few studies reported associations for milk intake by fat content, future studies need to investigate and report such associations.

It is interesting that we observed a statistically significant positive association of regular-fat dairy intake with late stage, but not a high-Gleason score, both of which are considered advanced prostate cancer. This statistically significant association is consistent with only one previous study, the Health Professional Follow-up Study [31]. This study is similar to ours by including butter in dairy product intake, but not in terms of having health professionals reflective of high socioeconomic status in their study. Our finding is also corroborated by the hypothesized biological mechanism linking androgen levels and fat intake to promoting prostate carcinogenesis [11,12], although we cannot exclude the possibility of a chance finding. Future studies may need to stratify prostate cancer by each of the prostate cancer characteristics to further elucidate etiology of dietary factors in prostate cancer.

The strengths of our study are the large sample size and comprehensive set of dairy products and potential confounders measured prospectively. This allowed us to assess dairy product intakes by fat content and fermentation methods and adjust for many potential confounding factors in our models. As part of a cancer screening trial, prostate cancer was ascertained prospectively and verified pathologically, allowing detailed information on prostate cancer diagnosis to be available. Hence, we were able to classify prostate cancer cases based on clinical stage and Gleason scores. In addition, we were able to adjust both prostate cancer screening frequency and results as confounders, which is in contrast to many previous studies that did not adjust for these important confounders [7]. We also had a sufficient number of cases to conduct stratified analyses.

Limitations in our study are possible misclassifications of dairy intake due to measurement errors inherent in self-reported dietary intake data often used in nutritional epidemiology [20]. Hence, we used energy-adjusted dairy intake (grams per 1000 kcal) rather than daily dairy intake to lessen the effects of these errors. Due to the prospective nature of our dietary assessments, differential misclassifications due to recall bias between cases and controls are unlikely. Recently, Mendelian randomization approach to use lactase genotype (*LCT*) as a proxy for milk or dairy intake was applied in two studies reporting a non-significant positive association of *LCT* genotype with prostate cancer risk, although it was significantly positively associated with milk and dairy products in both studies [32,33]. More future studies need to apply this approach and clarify discrepant associations of *LCT* genotype with milk or dairy intake and with prostate cancer risk. Another limitation was that the majority of participants were non-Hispanic white. Therefore, the generalizability of our findings to men in other ethnic groups is limited.

In conclusion, in this prospective analysis, dairy product consumption was not associated with prostate cancer overall, which does not support the harmful impact of dairy products on prostate cancer reported in previous studies. Our findings on the modest positive associations between 2%-fat milk intake and risk of advanced prostate cancer and between regular-fat dairy product intake and risk for late-stage prostate cancer, with no difference in the association by advanced status or clinical stage, respectively, need to be to be confirmed in other study populations.

## Figures and Tables

**Table 1:** Characteristics of the study population by quartiles of total dairy consumption *.

	Quartiles of Total Dairy Consumption			
	1	2	3	4
Number	12,368	12,368	12,368	12,368
Prostate cancer cases	960	1034	1069	1071
Age at interview	65.0 ± 5.6	65.6 ± 5.6	66.0 ± 5.8	66.5 ± 5.8
Non-Hispanic White	82.3%	91.1%	93.8%	95.5%
Frequency of prostate cancer screenings				
PSA	2.7 ± 2.8	2.8 ± 2.8	2.8 ± 2.8	2.7 ± 2.8
DRE	1.9 ± 1.9	1.9 ± 1.9	1.9 ± 1.9	1.9 ± 1.9
Maximum PSA levels	2.3 ± 2.4	2.3 ± 2.6	2.4 ± 2.5	2.5 ± 2.5
Education				
Completed high school or less	27.5%	25.3%	24.2%	25.1%
Post high-school training or some college	34.0%	32.3%	31.8%	32.1%
College graduation or more	38.5%	42.4%	44.0%	42.8%
Family history of cancer	51.6%	52.4%	52.2%	52.1%
Personal history of diabetes	7.7%	8.1%	7.9%	8.3%
Smoking status				
Never smokers	32.7%	36.4%	39.4%	42.1%
Current smokers	12.5%	9.4%	8.8%	9.6%
Former smokers	54.8%	54.2%	51.8%	48.3%
BMI (kg/m^2^)	27.6 ± 4.2	27.7 ± 4.2	27.5 ± 4.0	27.4 ± 4.0
<25 kg/m^2^	26.2%	24.5%	25.4%	25.8%
25–29.55 kg/m^2^	50.3%	51.4%	52.2%	52.9%
>30 kg/m^2^	23.5%	24.1%	22.4%	21.3%
Any physical activity	61.3%	64.6%	65.8%	64.6%
Any ibuprofen use	23.3%	23.5%	23.1%	22.1%
Alcohol consumption				
Never drinker	7.2%	7.3%	8.2%	11.4%
Former drinker	14.6%	13.6%	15.1%	16.6%
Current drinker	78.2%	79.1%	76.7%	72.0%
Total energy (kcal/day)	2008 ± 888	2053 ± 831	1972 ± 763	1946 ± 766

* Continuous variables presented as mean ± standard deviations. Abbreviations: PSA, prostate-specific antigen; DRE, digital rectal examination; BMI, body mass index.

**Table 2:** Risk of prostate cancer by quartiles of dairy product intakes *.

	Quartiles	Intake	Cases	HR	95% CI	*P*-Trend
Total dairy products	1 (lowest)	<47.2	960		Reference	
(g/1000 kcal)	2	47.2–101.3	1034	1.07	0.97, 1.16	
	3	101.3–194.7	1069	1.08	0.98, 1.17	
	4 (highest)	>194.7	1071	1.05	0.96, 1.15	0.48
Low-fat dairy products	1	<21.2	948		Reference	
(g/1000 kcal)	2	21.2–76.7	1041	1.08	0.98, 1.18	
	3	76.7–169.7	1075	1.09	1.00, 1.20	
	4	>169.7	1070	1.07	0.98, 1.17	0.34
Regular-fat dairy products	1	<3.1	975		Reference	
(g/1000 kcal)	2	3.1–9.2	1085	1.11	1.02, 1.21	
	3	9.2–21.7	1032	1.05	0.96, 1.15	
	4	>21.7	1042	1.05	0.96, 1.15	0.90
Fermented dairy products	1	<2.9	1000		Reference	
(g/1000 kcal)	2	2.9–7.3	1052	1.02	0.94, 1.11	
	3	7.3–15.8	1022	0.98	0.90, 1.07	
	4	>15.8	1060	1.01	0.93, 1.10	0.92
Non-fermented dairy products	1	<33.8	971		Reference	
(g/1000 kcal)	2	33.8–85.5	993	1.02	0.93, 1.12	
	3	85.5–178.4	1082	1.09	0.99, 1.19	
	4	>178.4	1088	1.07	0.98, 1.17	0.15

* Adjusted for age, race, Prostate, Lung, Colorectal and Ovarian (PLCO) Cancer Screening trial study center, PLCO trial arm, frequency of prostate cancer screening during the follow-up period, maximum PSA level during follow-up period, and family history of any cancer.

**Table 3:** Risk of prostate cancer by milk intake *.

	Quartiles	Intake	Cases	HR	95% CI
Total milk ^†^	1 (lowest)	<21.8	962		Reference
(g/1000 kcal)	2	21.8–71.9	1019	1.05	0.96, 1.14
	3	71.9–163.9	1088	1.10	1.01, 1.21
	4 (highest)	>163.9	1065	1.06	0.97, 1.15
1% fat milk	None	0	3385		Reference
(g/1000 kcal)	<median	0–83.2	361	0.99	0.89, 1.11
	≥median	>83.2	388	1.02	0.92, 1.14
2% fat milk	None	0	2711		Reference
(g/1000 kcal)	<median	0–59.3	688	1.02	0.93, 1.11
	≥median	>59.3	735	1.06	0.98, 1.15
Skim/nonfat/0.5% fat milk	None	0	2623		Reference
(g/1000 kcal)	<median	0–89.5	740	0.98	0.90, 1.07
	≥median	>89.5	771	1.00	0.92, 1.09
Whole milk	None	0	3633		Reference
(g/1000 kcal)	<median	0–37.0	239	0.90	0.79, 1.03
	≥median	>37.0	262	1.00	0.88, 1.13

* Adjusted for age, race, PLCO study center, PLCO trial arm, frequency of prostate cancer screening during the follow-up period, maximum PSA level during follow-up period, and family history of any cancer. ^†^ *P*-trend = 0.39 for the association between total milk and prostate cancer risk.

**Table 4:** Risk of prostate cancer by quartiles of dairy product intake stratified by advanced or non-advanced prostate cancer *.

	Quartiles	Cases	HR	95% CI	*P*-Trend
Non-advanced prostate cancer					
Total dairy products	1 (lowest)	476		Reference	
(g/1000 kcal)	2	528	1.09	0.96, 1.24	
	3	545	1.09	0.96, 1.24	
	4 (highest)	559	1.09	0.96, 1.23	0.35
Low-fat dairy products	1	467		Reference	
(g/1000 kcal)	2	528	1.10	0.97, 1.25	
	3	552	1.12	0.99, 1.28	
	4	561	1.12	0.99, 1.27	0.19
Regular-fat dairy products	1	486		Reference	
(g/1000 kcal)	2	578	1.17	1.03, 1.32	
	3	531	1.07	0.94, 1.21	
	4	513	1.03	0.90, 1.16	0.46
Fermented dairy products	1	492		Reference	
(g/1000 kcal)	2	527	1.02	0.90, 1.16	
	3	543	1.04	0.92, 1.17	
	4	546	1.03	0.91, 1.17	0.67
Non-fermented dairy products	1	483		Reference	
(g/1000 kcal)	2	500	1.03	0.91, 1.17	
	3	562	1.13	0.99, 1.28	
	4	563	1.09	0.97, 1.24	0.17
Advanced prostate cancer					
Total dairy products	1	478		Reference	
	2	501	1.04	0.92, 1.18	
(g/1000 kcal)	3	515	1.06	0.93, 1.20	
	4	506	1.02	0.90, 1.16	0.92
Low-fat dairy products	1	473		Reference	
(g/1000 kcal)	2	508	1.06	0.94, 1.20	
	3	516	1.07	0.94, 1.21	
	4	503	1.03	0.90, 1.17	0.92
Regular-fat dairy products	1	481		Reference	
(g/1000 kcal)	2	504	1.06	0.93, 1.20	
	3	497	1.04	0.92, 1.18	
	4	518	1.07	0.94, 1.21	0.43
Fermented dairy products	1	500		Reference	
(g/1000 kcal)	2	517	1.02	0.90, 1.15	
	3	473	0.93	0.81, 1.05	
	4	510	1.00	0.88, 1.13	0.89
Non-fermented dairy products	1	483		Reference	
(g/1000 kcal)	2	487	1.01	0.89, 1.14	
	3	511	1.05	0.92, 1.19	
	4	519	1.04	0.92, 1.19	0.49

* Adjusted for age, race, PLCO study center, PLCO trial arm, frequency of prostate cancer screening during the follow-up period, maximum PSA level during follow-up period, and family history of any cancer. Non-advanced status was defined as cases who had both clinical stages I/II and Gleason scores of <7; advanced status was defined as cases who had clinical stages III/IV and/or Gleason scores of ≥7.

**Table 5:** Risk of prostate cancer by milk intake stratified by advanced or non-advanced prostate cancer *.

	Quartiles	Cases	HR	95% CI
Non-advanced prostate cancer				
Total milk ^†^	1 (lowest)	480		Reference
(g/1000 kcal)	2	510	1.05	0.93, 1.19
	3	562	1.13	1.00, 1.28
	4 (highest)	556	1.09	0.96, 1.23
1% fat milk	None	1735		Reference
(g/1000 kcal)	<median	179	0.95	0.81, 1.11
	≥median	194	0.98	0.85, 1.14
2% fat milk	None	1404		Reference
(g/1000 kcal)	<median	345	0.99	0.88, 1.11
	≥median	359	0.99	0.88, 1.11
Skim/nonfat/0.5% fat milk	None	1291		Reference
(g/1000 kcal)	<median	400	1.09	0.97, 1.22
	≥median	417	1.10	0.98, 1.23
Whole milk	None	1856		Reference
(g/1000 kcal)	<median	115	0.86	0.71, 1.04
	≥median	137	1.03	0.87, 1.23
Advanced prostate cancer				
Total milk ^†^	1 (lowest)	475		Reference
(g/1000 kcal)	2	505	1.05	0.93, 1.19
	3	517	1.07	0.95, 1.22
	4 (highest)	503	1.03	0.90, 1.17
1% fat milk	None	1631		Reference
(g/1000 kcal)	<median	179	1.03	0.89, 1.21
	≥median	190	1.06	0.91, 1.24
2% fat milk	None	1288		Reference
(g/1000 kcal)	<median	339	1.05	0.93, 1.18
	≥median	373	1.14	1.02, 1.28
Skim/nonfat/0.5% fat milk	None	1312		Reference
(g/1000 kcal)	<median	338	0.88	0.78, 1.00
	≥median	350	0.91	0.81, 1.02
Whole milk	None	1754		Reference
(g/1000 kcal)	<median	123	0.95	0.79, 1.14
	≥median	123	0.96	0.80, 1.15

* Adjusted for age, race, PLCO study center, PLCO trial arm, frequency of prostate cancer screening during the follow-up period, maximum PSA level during follow-up period, and family history of any cancer. Non-advanced status was defined as cases who had both clinical stages I/II and Gleason scores of <7; advanced status was defined as cases who had clinical stages III/IV and/or Gleason scores of ≥7. ^†^ *P*-trend = 0.25 for the association between total milk intake and non-advanced prostate cancer risk and 0.92 for the association between total milk intake and advanced prostate cancer risk.

## Supplementary Materials

The following are available online at https://www.mdpi.com/2072-6643/11/7/1615/s1, Table S1: Dairy product items included in dairy product subgroups, Table S2. Risk of prostate cancer by quartiles of dairy product intakes stratified by the clinical stage of prostate cancer, Table S3. Risk of prostate cancer by quartiles of dairy product intakes stratified by Gleason score.
