# Supplementary material for: Dairy Product Consumption and Prostate Cancer Risk in the United States
Source: Nutrients. 2019 Jul 16;11(7):1615. doi: 10.3390/nu11071615 (PMC6683061; doi:10.3390/nu11071615)
Supplement: Supplementary file 1 [file nutrients-11-01615-s001.pdf]

Supplementary Table 1. Dairy product items included in dairy product subgroups

Subgroups	Dairy product items
Regular-fat dairy products	Whole milk, sour cream, ice cream, several cheeses, and regular butter.
Low-fat dairy products	Nonfat/skim/low-fat milk, low-fat sour cream, nonfat/low-fat cream cheese, nonfat/low-fat cheese, low-fat ice cream or frozen yogurt, yogurt, and reduced-fat butter.
Fermented products	Yogurt, several cheeses, and sour cream.
Non-fermented products	Milk, cream, ice cream and butter.

Supplementary Table 2. Risk of prostate cancer by quartiles of dairy product intakes stratified by the clinical stage of prostate cancer*

	Quartiles	Cases	HR	95% CI	*P*-trend
Early stage (Stage I/II)					
Total dairy products	1 (lowest)	874		Reference	
(g/1000 kcal)	2	937	1.06	0.96, 1.16	
	3	952	1.05	0.95, 1.15	
	4 (highest)	963	1.04	0.94, 1.14	0.75
Low-fat dairy products	1	847		Reference	
(g/1000 kcal)	2	947	1.09	0.99, 1.20	
	3	965	1.10	0.99, 1.20	
	4	967	1.08	0.98, 1.19	0.35
Regular-fat dairy products	1	888		Reference	
(g/1000 kcal)	2	984	1.11	1.01, 1.21	
	3	934	1.04	0.95, 1.15	
	4	920	1.02	0.93, 1.12	0.54
Fermented dairy products	1	909		Reference	
(g/1000 kcal)	2	941	1.00	0.92, 1.10	
	3	916	0.97	0.88, 1.06	
	4	960	1.01	0.92, 1.11	0.83
Non-fermented dairy products	1	885		Reference	
(g/1000 kcal)	2	887	1.00	0.91, 1.10	
	3	978	1.08	0.98, 1.18	
	4	976	1.05	0.95, 1.15	0.28
Late stage (III/IV)					
Total dairy products	1	86		Reference	
	2	97	1.15	0.86, 1.54	
(g/1000 kcal)	3	116	1.36	1.03, 1.81	
	4	108	1.24	0.93, 1.66	0.20
Low-fat dairy products	1	101		Reference	
(g/1000 kcal)	2	94	0.92	0.69, 1.22	
	3	109	1.07	0.81, 1.41	
	4	103	0.99	0.75, 1.32	0.86
Regular-fat dairy products	1	87		Reference	
(g/1000 kcal)	2	100	1.12	0.84, 1.49	
	3	98	1.09	0.82, 1.46	

	4	122	1.37	1.04, 1.82	0.02
Fermented dairy products	1	90		Reference	
(g/1000 kcal)	2	111	1.19	0.90, 1.58	
	3	106	1.11	0.84, 1.48	
	4	100	1.05	0.78, 1.40	0.78
Non-fermented dairy products	1	86		Reference	
(g/1000 kcal)	2	106	1.26	0.95, 1.68	
	3	103	1.22	0.91, 1.63	
	4	112	1.29	0.97, 1.73	0.19

*Adjusted for age, race, PLCO study center, PLCO trial arm, frequency of prostate cancer screening during the follow-up period, maximum PSA level during follow-up period, and family history of any cancer.

Supplementary Table 3. Risk of prostate cancer by quartiles of dairy product intakes stratified by Gleason score*

	Quartiles	Cases	HR	95% CI	*P*-trend
Low Gleason score (<7)					
Total dairy products	1 (lowest)	486		Reference	
(g/1000 kcal)	2	539	1.09	0.96, 1.23	
	3	563	1.10	0.98, 1.25	
	4 (highest)	576	1.10	0.97, 1.24	0.26
Low-fat dairy products	1	477		Reference	
(g/1000 kcal)	2	539	1.10	0.97, 1.24	
	3	569	1.13	1.00, 1.28	
	4	579	1.13	0.99, 1.28	0.13
Regular-fat dairy products	1	501		Reference	
(g/1000 kcal)	2	589	1.15	1.02, 1.30	
	3	549	1.07	0.95, 1.21	
	4	525	1.02	0.90, 1.15	0.43
Fermented dairy products	1	506		Reference	
(g/1000 kcal)	2	547	1.03	0.91, 1.16	
	3	553	1.02	0.91, 1.16	
	4	558	1.02	0.91, 1.16	0.85
Non-fermented dairy products	1	494		Reference	
(g/1000 kcal)	2	511	1.03	0.91, 1.17	
	3	580	1.14	1.01, 1.29	
	4	579	1.10	0.97, 1.24	0.13
High Gleason score (≥7)					
Total dairy products	1	466		Reference	
	2	484	1.04	0.91, 1.18	
(g/1000 kcal)	3	495	1.04	0.92, 1.19	
	4	483	1.00	0.88, 1.14	0.88
Low-fat dairy products	1	458		Reference	
(g/1000 kcal)	2	493	1.07	0.94, 1.21	
	3	496	1.06	0.93, 1.21	
	4	481	1.02	0.89, 1.16	0.93
Regular-fat dairy products	1	463		Reference	
(g/1000 kcal)	2	492	1.08	0.95, 1.22	
	3	477	1.04	0.91, 1.18	

	4	496	1.07	0.94, 1.21	0.53
Fermented dairy products	1	480		Reference	
(g/1000 kcal)	2	495	1.02	0.90, 1.16	
	3	457	0.94	0.82, 1.07	
	4	496	1.01	0.89, 1.15	0.86
Non-fermented dairy products	1	471		Reference	
(g/1000 kcal)	2	469	0.99	0.88, 1.13	
	3	492	1.04	0.91, 1.18	
	4	496	1.03	0.90, 1.17	0.64

*Adjusted for age, race, PLCO study center, PLCO trial arm, frequency of prostate cancer screening during the follow-up period, maximum PSA level during follow-up period, and family history of any cancer.
